# Fumonisin B_1_ Epigenetically Regulates PTEN Expression and Modulates DNA Damage Checkpoint Regulation in HepG2 Liver Cells

**DOI:** 10.3390/toxins12100625

**Published:** 2020-09-30

**Authors:** Thilona Arumugam, Terisha Ghazi, Anil Chuturgoon

**Affiliations:** Discipline of Medical Biochemistry and Chemical Pathology, School of Laboratory Medicine and Medical Sciences, College of Health Sciences, George Campbell Building, Howard College, University of KwaZulu-Natal, Durban 4041, South Africa; cyborglona@gmail.com (T.A.); terishaghazi@gmail.com (T.G.)

**Keywords:** Fumonisin B_1_, DNA damage, epigenetics, PTEN, H3K4me3, Checkpoint Kinase 1

## Abstract

Fumonisin B_1_ (FB_1_), a *Fusarium*-produced mycotoxin, is found in various foods and feeds. It is a well-known liver carcinogen in experimental animals; however, its role in genotoxicity is controversial. The current study investigated FB_1_-triggered changes in the epigenetic regulation of PTEN and determined its effect on DNA damage checkpoint regulation in human liver hepatoma G2 (HepG2) cells. Following treatment with FB_1_ (IC_50_: 200 µM; 24 h), the expression of miR-30c, KDM5B, PTEN, H3K4me3, PI3K, AKT, p-ser473-AKT, CHK1, and p-ser280-CHK1 was measured using qPCR and/or Western blot. H3K4me3 enrichment at the PTEN promoter region was assayed via a ChIP assay and DNA damage was determined using an ELISA. FB_1_ induced oxidative DNA damage. Total KDM5B expression was reduced, which subsequently increased the total H3K4me3 and the enrichment of H3K4me3 at PTEN promoters. Increased H3K4me3 induced an increase in PTEN transcript levels. However, miR-30c inhibited PTEN translation. Thus, PI3K/AKT signaling was activated, inhibiting CHK1 activity via phosphorylation of its serine 280 residue preventing the repair of damaged DNA. In conclusion, FB_1_ epigenetically modulates the PTEN/PI3K/AKT signaling cascade, preventing DNA damage checkpoint regulation, and induces significant DNA damage.

## 1. Introduction

Fumonisins are major food-borne mycotoxins produced by fungi belonging to the *Fusarium* genus [1,2]. Presently, 28 fumonisin homologues have been characterized into the following groups: fumonisins A, B, C, and P [2]. Over 70% of fumonisins produced are fumonisin B_1_ (FB_1_), making it the most prevalent and toxicologically relevant homologue [3].

FB_1_ contamination is common in maize and cereal-related products in several countries throughout the world, with concentrations reaching as high as 30,000 µg/kg [4]. Poor food processing, handling, and storage conditions aide FB_1_ contamination, thereby increasing the risk of exposure for both animals and humans [5]. The effect of FB_1_ in animals is sex-dependent and has species-specific toxicity, with the liver, kidney, and nervous system being the most common targets [6,7,8,9,10,11]. The International Agency for Research on Cancer (IARC) has classified FB_1_ as a class 2B carcinogen [12]. Studies on rodents have demonstrated that FB_1_ can initiate and promote cancer [1,13], while the consumption of FB_1_-contaminated commodities has been associated with increased incidence of hepatocellular and/or esophageal carcinomas [14,15]. Earlier studies have dismissed FB_1_ as a mutagen and reported that FB_1_ is a weak genotoxin [16] or that it showed no signs of genotoxicity [17,18]. Irrespective of these earlier studies, numerous studies have since observed that a consequence of FB_1_ exposure is extensive DNA damage through strand breaks, micronuclei induction, and fragmentation [19,20,21].

Cells are equipped with a complex network of DNA damage responses (DDRs) that coordinate DNA repair and consequently cell fate [22]. The tumor suppressor phosphatase and tensin homolog (PTEN) controls multiple cellular processes including growth and differentiation by opposing the phosphoinositide 3-kinases (PI3K)/protein kinase B (AKT) signaling cascade [23,24]. Emerging evidence has demonstrated the unique role PTEN plays in maintaining genomic stability and DNA repair [25,26]. PTEN responds to DNA damage by inhibiting the PI3K/AKT cascade and preventing the inhibitory phosphorylation of checkpoint kinase 1 (CHK1). This activates checkpoint regulation and induces cell cycle arrest, which allows for the repair of DNA [27,28]. Underlining the important role of PTEN, poor expression of PTEN is a common risk factor in the occurrence of liver pathologies [29,30]. Studies have elucidated that poor expression of PTEN may be due to epigenetic alterations [31].

Disruption to the epigenome may contribute to poor PTEN expression. Small non-coding RNAs, known as microRNAs (miRNA), such as miR-19a and miR-21, reduce PTEN gene expression by binding to the 3′ untranslated region (3′UTR) of *PTEN* mRNA and inhibiting its translation [32,33], while the trimethylation of lysine 4 residues of histone 3 (H3K4me3) on the promoter region of *PTEN* is associated with active transcription [34].

While the role of PTEN in cellular functioning has been well established, further research should be undertaken to determine the epigenetic mechanisms in which PTEN is regulated. Moreover, the epigenetic effects of FB_1_ in humans have only recently begun to be uncovered and no study to date has determined the effects FB_1_ has on PTEN [21,35]. Previously, Chuturgoon et al. [35] conducted miRNA profile arrays in human hepatoma G2 (HepG2) cells following FB_1_ exposure and found miR-30c to be one of the major miRNAs affected. Through computational prediction analysis, we found a possible link between miR30c, PTEN, and the histone lysine demethylase 5B (KDM5B). KDM5B catalyzes the removal of methyl groups from histone 3 lysine 4 (H3K4) [36]. H3K4me3 is predominantly found at transcriptional start sites, where it promotes gene transcription [37]. Therefore, we proposed that together miR-30c and KDM5B mediate the epigenetic regulation of PTEN. The current study determined the consequences of FB_1_ exposure on DNA damage and DNA damage checkpoint regulation via the PTEN/PI3K/AKT network. Further, we determined FB_1_ epigenetic regulation of PTEN via miR-30c and H3K4me3 in human liver (HepG2) cells.

## 2. Results

### 2.1. FB_1_ Induces DNA Damage in HepG2 Cells

**Figure 1:**
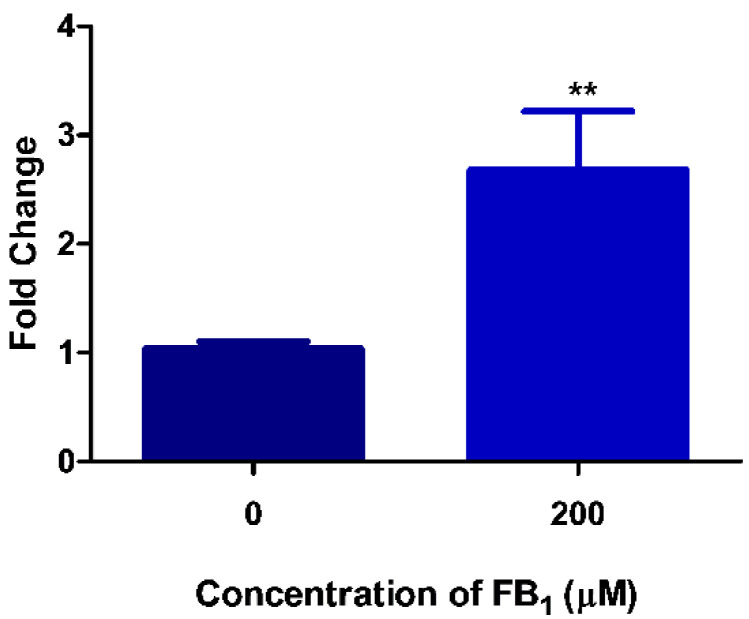
Fumonisin B_1_ (FB_1_) significantly increased the oxidative DNA damage biomarker, 8-OHdG, in human hepatoma G2 (HepG2) cells (** *p* < 0.01).

FB_1_ negatively impacts redox homeostasis, which results in oxidative damage to cellular structures. We assessed FB_1_-mediated DNA damage by evaluating levels of the oxidative DNA damage biomarker—8-hydroxy-2′-deoxyguanosine (8-OHdG). FB_1_ significantly increased the level of 8-OHdG (2.68-fold) compared with the control (*p* = 0.0061; Control: 1.04 ± 0.0641 vs. FB_1_: 2.68 ± 0534; Figure 1).

### 2.2. FB_1_ Increases miR-30c Expression in HepG2 Cells

**Figure 2:**
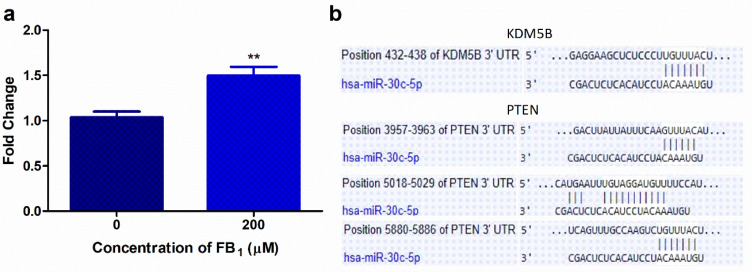
The effect of FB_1_ on miR-30c levels in HepG2 cells and potential miR-30c targets. (**a**) FB_1_ significantly elevated miR-30c expression (** *p* ≤ 0.01). (**b**) Target Scan analysis of miR-30c with the 3′ untranslated region (3′UTR) of *KDM5B* and *PTEN*.

Since PTEN initiates DNA damage responses and miR-30c has been shown to disrupt DNA damage responses, we investigated the epigenetic regulation of PTEN [26,38]. miR-30c is involved in regulating cell cycle transition, proliferation, and lipid metabolism. FB_1_ (IC_50_; 200 µM) significantly upregulated miR-30c by 1.47-fold (*p* = 0.0023; Control: 1.04 ± 0.0642 vs. FB_1_ 1.47 ± 0.149; Figure 2a).

Target Scan version 7.2 (http://www.targetscan.org/vert_72/) was used to identify putative mRNA targets of miR-30c. miR-30c has complimentary base pairs with *PTEN* (at positions 3957–3963, 5018–5029, and 5880–5886 in the 3′UTR) and *KDM5B* (at positions 432–438 in the 3′UTR) (Figure 2b)

### 2.3. FB_1_ Induces H3K4me3 by Downregulating KDM5B in HepG2 Cells

**Figure 3:**
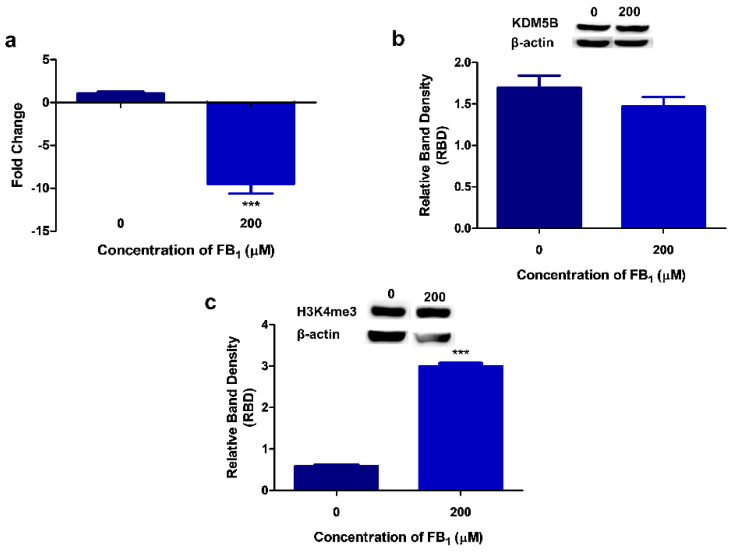
The effect of FB_1_ on KDM5B and H3K4me3 levels in HepG2 cells. FB_1_ reduced both the transcript ((**a**); *** *p* ≤ 0.0001) and protein ((**b**); *p* > 0.05) expression of KDM5B. This may have led to the subsequent increase in total H3K4me3 ((**c**); *** *p* ≤ 0.0001).

Since FB_1_ altered the expression of miR-30c (which has a complimentary sequence to KDM5B 3’ UTR), we evaluated the gene and protein expression of KDM5B. FB_1_ decreased *KDM5B* transcript levels by 9.86-fold (*p* < 0.0001; Control: 1.04 ± 0.0642 vs. FB_1_: 9.86 ± 1.15; Figure 3a). KDM5B protein expression (Figure 3b) was reduced slightly (*p* = 0.2966) by FB_1_ (1.47 ± 0.117 RBD) in comparison with the control (1.70 ± 0.142 RBD).

KDM5B is a negative regulator of H3K4me3; hence, we determined the effect of FB_1_ on H3K4me3. FB_1_ (3.00 ± 0.0589 RBD) induced a considerable increase (*p* < 0.0001) in total H3K4me3 compared with the control (0.585 ± 0.00423 RBD; Figure 3c).

### 2.4. FB_1_ Alters PTEN Expression in HepG2 Cells

**Figure 4:**
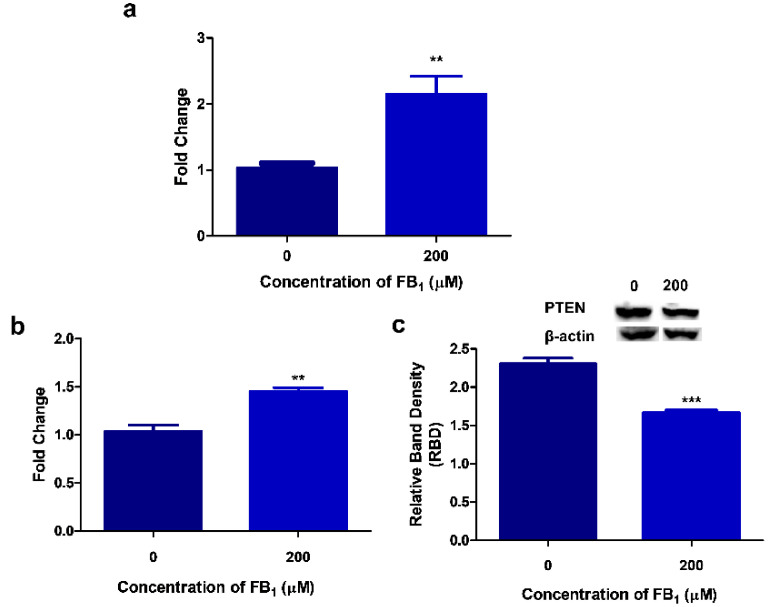
FB_1_-induced KDM5B and miR-30c modulates PTEN expression. PTEN expression is influenced by both KDM5B and miR-30c. FB_1_ increased H3K4me3 at *PTEN* promoter regions ((**a**); ** *p* < 0.01), which resulted in significantly higher levels of PTEN transcripts ((**b**); ** *p* < 0.01). However, miR-30c negatively influenced PTEN translation/protein expression ((**c**); *** *p* < 0.0001).

PTEN expression may be influenced by KDM5B and miR-30c. In addition to the total H3K4me3 levels, FB_1_ also induced a significant 2.5-fold upregulation of H3K4me3 at *PTEN* promoter regions (*p* = 0.0052; Control: 1.04 ± 0.0641 vs. FB_1_: 2.15 ± 0.273; Figure 4a).

H3K4me3 at promoter regions is associated with active transcription. The FB_1_-induced increase in H3K4me3 corresponded with active transcription of the *PTEN* gene with a 1.46-fold increase (*p* = 0.0039; Control: 1.04 ± 0.0641 vs. FB_1_: 1.46 ± 0.0354; Figure 4b). However, PTEN protein expression was significantly downregulated (*p* = 0.0001) by FB_1_ (1.67 ± 0.0110 RBD) compared with the control (2.31 ± 0.0749 RBD; Figure 4c).

### 2.5. FB_1_ Affects PI3K/AKT Signaling in HepG2 Cells

**Figure 5:**
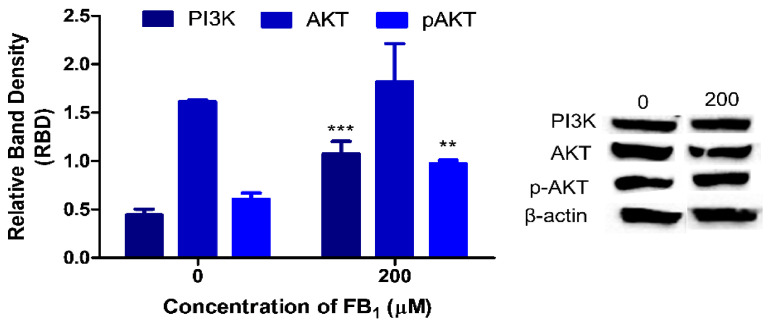
The effect of FB_1_ on the PI3K/AKT signaling cascade. The protein expression of PI3K, AKT, and pAKT in HepG2 cells was evaluated using Western blotting. FB_1_ increased PI3K (*** *p* < 0.0001), AKT (*p* > 0.05), and p-ser473-AKT (** *p* < 0.01) protein expression. PI3K and AKT expression was normalized against β-actin, and p-ser473-AKT was normalized against AKT.

Numerous biological processes are regulated by the PTEN/PI3K/AKT signaling network. PI3K protein expression (*p* = 0.0014; Figure 5) was 2.44-fold greater in FB_1_-exposed cells (1.08 ± 0.126 RBD) compared with the control (0.443 ± 0.0600 RBD).

Total AKT protein expression was slightly increased (*p* = 0.4200; Figure 5) by FB_1_ (Control 1.61 ± 0.0148 RBD vs. FB_1_ 1.82 ± 0.396 RBD). AKT is activated by the phosphorylation of serine 473 within the carboxy terminus. FB_1_ significantly increased the phosphorylation of AKT (*p* = 0.001, 0.973 ± 0.0350 RBD; Figure 5) compared with the control (0.604 ± 0.0661 RBD).

### 2.6. FB_1_ Modulates CHK1 Expression and Activity in HepG2 Cells

**Figure 6:**
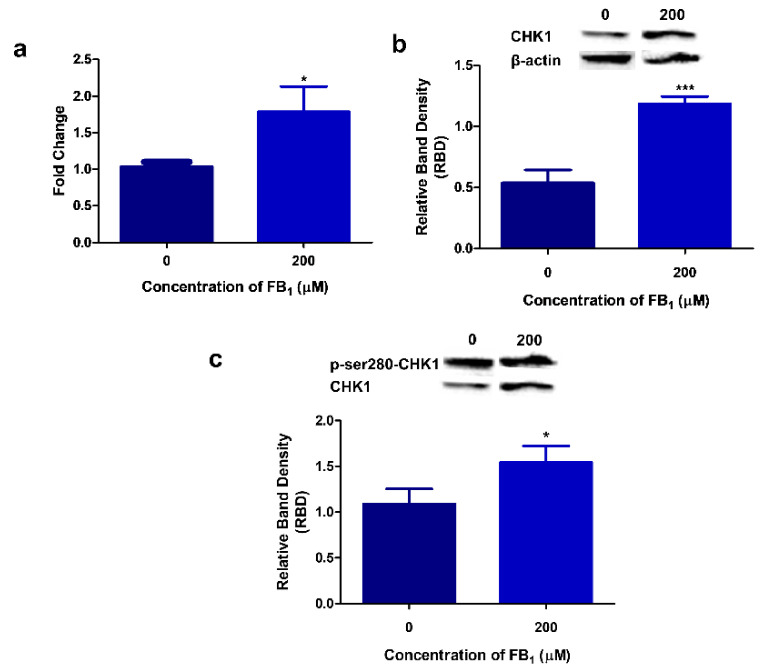
The effect of FB_1_ on CHK1 expression. FB_1_ significantly increased *CHK1* transcript levels ((**a**); * *p* < 0.05), CHK1 protein expression ((**b**); *** *p* < 0.0001), and p-ser280-CHK1 ((**c**); * *p* < 0.05). CHK1 expression was normalized against β-actin and p-ser280-CHK1 was normalized against CHK1.

CHK1 is critical in coordinating DDR and cell cycle checkpoints. FB_1_ elevated *CHK1* transcript levels by 1.79 (*p* = 0.0209; Figure 6a). Western blotting revealed an increase in total CHK1 protein expression (*p* = 0.0008; Control 0.540 ± 0.105 RBD vs. FB_1_ 1.18 ± 0.0614 RBD; Figure 6b). Active PI3K/AKT signaling phosphorylates serine 280 of CHK1 and inactivates it. FB_1_ significantly elevated (*p* = 0.0314; 1.54 ± 0.179 RBD) p-ser280-CHK1 expression in comparison with the control (1.09 ± 0.162 RBD; Figure 6c). This suggests that FB_1_ inactivates CHK1 via the PI3K/AKT signaling pathway.

## 3. Discussion

Considering that FB_1_ contamination of agricultural products is common throughout the world, it is necessary to evaluate the health hazards FB_1_ poses to humans and animals. Several studies have attributed oxidative stress as one of the mechanisms in which FB_1_ exerts its toxicity [39,40,41,42,43]. Excessive production of reactive oxygen species (ROS) results in oxidative damage to cells and macromolecules including DNA [44]. While some studies have disputed the genotoxic potential of FB_1_ [17,18], others have reported chromosomal aberrations and oxidative DNA damage triggered by FB_1_ exposure [16,39,45,46]. Apart from inducing DNA damage, FB_1_ may disrupt DDR network and repair processes. One potential mechanism could be through the PTEN/PI3K/AKT/CHK1 axis.

To better understand the genotoxic potential of FB_1_, we set out to determine if FB_1_ induces DNA damage and if it alters DNA damage checkpoint regulation via the PTEN/PI3K/AKT/CHK1 network. Seeing that poor PTEN expression is common in toxicity, we further determined the effects of FB_1_ on the epigenetic regulation of PTEN via miR-30c and H3K4me3 in human hepatoma G2 (HepG2) cells. The liver is one of the primary organs in which FB_1_ is thought to accumulate, and is usually the initial site for the metabolism and detoxification of food and food contaminants [47,48]. Due to the limitations of primary hepatocytes such as poor availability, short life span, inter-donor variability, loss of hepatic function, and early phenotypic changes, we opted to use the HepG2 cell line for this study [49,50]. The DNA of HepG2 cells is less sensitive to damage caused by xenobiotics than intact hepatocytes [51,52]. Moreover, no mutations have been found in the PTEN gene of the HepG2 cell line, making it an apt model for testing genotoxicity and epigenetic changes that may occur as a result of FB_1_ exposure [53]. The effect of FB_1_ on HepG2 cell viability was conducted using a crystal violet assay in accordance with Feoktistova et al. [54] (Supplementary Figure S1). FB_1_ reduced HepG2 cell viability in a dose-dependent manner (5, 50, 100, 200 µM). For subsequent assays, HepG2 cells were exposed to 5, 100, and 200 µM FB_1_ as they represented 90%, 70%, and 50% cell viabilities, respectively. Results obtained for 5 and 100 µM can be found in the Supplementary Materials (Supplementary Figures S2–S7).

We evaluated the genotoxic potential of FB_1_ by determining if FB_1_ inflicted damage on DNA. Previously, we showed that at 200 µM FB_1_ enhanced ROS production, resulting in oxidative stress [43]. Thus, in the present study we measured 8-OHdG levels as a marker of oxidative DNA damage. The low redox potential of guanine makes it the most vulnerable base and its product (8-OHdG) the best characterized oxidative lesion [55]. We found a significant 2.63-fold increase in 8-OHdG levels in the DNA of FB_1_-exposed cells (Figure 1). The incorporation of 8-OHdG into DNA can generate double strand breaks, making this a harmful lesion [56]. Several other in vivo and in vitro studies observed DNA fragmentation as a consequence of FB_1_ exposure, proving that FB_1_ is genotoxic [19,20,21,40].

While the impact FB_1_ has on DNA damage has been thoroughly researched, little is known on the impact it may have on DNA damage responses. Hence, we investigated the effect of FB_1_ on the PTEN/PI3K/AKT/CHK1 axis and further determined if FB_1_ effects the epigenetic regulation of PTEN. Currently, only a few studies have demonstrated the effects of FB_1_ on epigenetic modifications in humans. Previously, Chuturgoon et al. (2014) screened for alterations in the miRNA expression profile of HepG2 cells exposed to 200 µM FB_1_. miR-30c was one of the miRNAs shown to be dysregulated [35]. MiR-30c is an important regulator of hepatic liver metabolism, apoptosis, cell cycle transition, proliferation, and differentiation [57,58,59]. We found that the expression of miR-30c was significantly increased after exposure to 200 µM FB_1_ (Figure 2a). Using an online computational prediction algorithm (TargetScan version 7.2), miR-30c was found to possibly target PTEN and KDM5B (Figure 2b). miRNAs silence their mRNA targets through mRNA cleavage or translational repression [60,61,62]. FB_1_ reduced KDM5B transcript and protein levels in HepG2 cells (Figure 3a,b). While FB_1_ reduced KDM5B mRNA levels by 9.86-fold, only a slight decrease in protein expression was observed. A previous study did find a minor increase in *KDM5B* transcript levels at 200 µM FB_1_; however, these results were not statistically significant [35]. Further studies using miR-30c inhibitors and mimics need to be conducted to validate miR-30c regulation of KDM5B expression.

FB_1_ can also induce epigenetic changes through the post-translational modifications of histones, but no study to date has investigated these changes in humans [63,64,65]. Here, we identified changes to H3K4 methylation. Although there was a slight decrease in KDM5B, we found a significant increase in global H3K4me3 (Figure 3c). H3K4me3 is predominantly found at transcriptional start sites, where it regulates the binding of transcription factors and activates gene transcription [66,67]. Thus, we determined H3K4me3 levels at the *PTEN* promoter region using the ChIP assay; FB_1_ significantly increased H3K4me3 at the *PTEN* promoter region (Figure 4a). These results correspond to the substantial elevation in *PTEN* transcript levels; however, the protein expression of PTEN was decreased (Figure 4b,c). PTEN may be post-transcriptionally regulated by miR-30c, as the decrease in PTEN protein expression corresponded to the increased miR-30c levels. Hence, miR-30c may act as a possible inhibitor of PTEN translation.

PTEN functions in regulating several cellular processes by antagonizing the PI3K/AKT signaling cascade [68]. Emerging evidence has revealed that PTEN is central in maintaining the DNA integrity by regulating DDR pathways via its interaction with CHK1 [27,28]. Additionally, PTEN regulates the activity of CHK1 via the PI3K/AKT axis [69,70,71,72]. Briefly, PTEN dephosphorylates the primary product of PI3K, phosphatidylinositol-3,4,5-triphosphate (PIP3). PIP3 activates AKT via its phosphorylation at serine residue 473 [69]. Downregulation of PTEN permitted PI3K/AKT signaling to proceed undisturbed as PI3K and p-ser473-AKT expression was upregulated (Figure 5). FB_1_ inhibits ceramide formation and promotes the formation of spingoid bases [73]. This may explain the activation of AKT by FB_1_, as ceramide inhibits PI3K and promotes the dephosphorylation of AKT on serine 473 [74,75]. Furthermore, sphingosine-1-phosphate activates PI3K/AKT signaling by binding to G_I_-coupled receptors [76].

AKT, in its activated form, inhibits CHK1 functioning by phosphorylating serine 280 of CHK1 [69,71,72]. Activated PI3K/AKT signaling impaired CHK1 function via increased p-ser-280-CHK1 after FB_1_ exposure (Figure 6). During DDR, CHK1 arrests cells at the G1/S, S, and G2/M phases by phosphorylating the cdc25 family of phosphatases [77,78]. This allows for DNA repair to occur prior to determining cell fate. Although we did not analyze changes in cell cycle, previous studies have shown that FB_1_ disrupts G1/S blockade; however, increased G2/M arrest was observed [79,80,81]. Nonetheless, the inhibitory phosphorylation of CHK1 coincided with DNA damage after FB_1_ exposure in HepG2 cells, as cell cycle checkpoints were disrupted, inhibiting repair.

In addition to 200 µM FB_1_, the effects of 5 and 100 µM FB_1_ were investigated (Supplementary Figures S2–S7). While cells exposed to 5 and 200 µM FB_1_ responded in a similar manner, the effect at 200 µM FB_1_ was exacerbated. Additionally, we observed that 100 µM FB_1_ generally had the opposite effect on 8-OHdG levels, H3K4 trimethylation on the PTEN promoter, and the expression of miR-30c, KDM5B, PTEN, PI3K, p-ser423-AKT, CHK1, and p-ser-280-CHK1 in HepG2 cells in comparison with the 5 and 200 µM FB_1_. As with many toxins, this suggests that FB_1_ is associated with a biphasic dose response [82].

## 4. Conclusions

**Figure 7:**
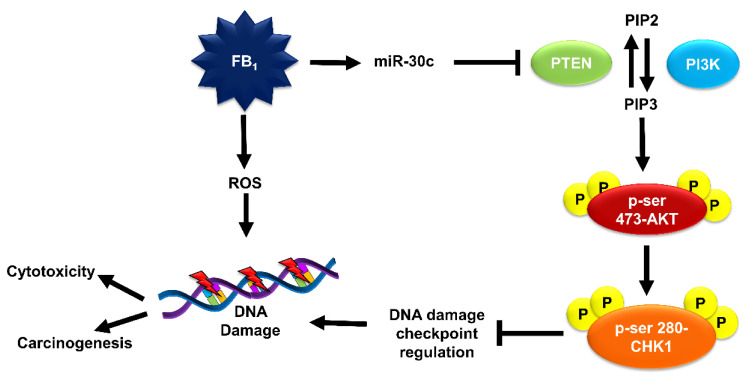
FB_1_ induces oxidative DNA damage. It further impairs DNA damage checkpoint regulation pathways via the PTEN/PI3K/AKT/CHK1 axis by epigenetically regulating PTEN. FB_1_ upregulates miR-30c, which inhibits PTEN translation, allowing for the phosphorylation of PIP2 to PIP3 by PI3K. This triggers the phosphorylation of AKT and subsequent phosphorylation of ser-280-CHK1, inhibiting CHK1 activity. Inhibition of CHK1 inhibits DNA damage checkpoint regulation. The resulted DNA damage may either contribute to FB_1_-mediated cytotoxicity or carcinogenicity.

This study further confirms the genotoxic potential of FB_1_, and that the inhibition of DNA damage checkpoint regulation may allow cells to evade DNA repair. FB_1_ epigenetically downregulates the expression of PTEN via miR-30c. The downregulation of PTEN inhibits DNA damage checkpoint regulation via the PI3K/AKT signaling network, preventing the repair of oxidative DNA lesions induced by FB_1_ (Figure 7)_._ Needless to say, further investigation should be conducted using miRNA inhibitors and mimics, and on whether the outcome of FB_1_-induced DNA damage and impaired DNA damage checkpoint regulation contributes to its cytotoxicity or carcinogenicity.

## 5. Method and Materials

### 5.1. Materials

FB_1_ *(Fusarium moniliforme*, 62580) was purchased from Cayman Chemicals (Ann Arbor, MI, USA). The HepG2 cell line (HB-8065) was procured from the American Type Culture Collection (ATCC). Cell culture consumables were purchased from Whitehead Scientific (Johannesburg, South Africa). Western blot reagents were obtained from Bio-Rad (Hercules, CA, USA). All other reagents were purchased from Merck (Boston, MA, USA), unless otherwise stated.

### 5.2. Cell Culture and Treatments

HepG2 cells (passage 3; 1.5 × 10^6^) were cultured in complete culture media (CCM: Eagle’s Minimum Essentials Medium (EMEM) supplemented with 10% fetal calf serum, 1% penicillin–streptomycin–fungizone, and 1% L-glutamine) at 37 °C in a 5% CO_2_ humidified incubator until 80% confluent. Thereafter, cells were treated with varying concentrations of FB_1_ (5, 100, and 200 µM) for 24 h. These FB_1_ concentrations were obtained from the crystal violet assay (Supplementary Figure S1) and represented 90%, 70%, and 50% cell viabilities, respectively. An untreated control was prepared along with the FB_1_ treatments. Data obtained using 200 µM FB_1_ (IC_50_) are shown in the main text. The results for all assays conducted using 5 and 100 µM FB_1_ are available in the Supplementary Material (Supplementary Figures S2–S7). Results were verified by performing two independent experiments in triplicate.

### 5.3. DNA Damage

DNA was isolated using the FlexiGene DNA isolation kit (Qiagen, Hilden, Germany, 512608). Extracted DNA was used to determine 8-OHdG levels using the DNA damage ELISA kit (Enzo Life Sciences, New York, NY, USA, ADI-EKS-350), as per the manufacturer’s instructions.

### 5.4. RNA Isolation and Quantitative Polymerase Chain Reaction (qPCR)

RNA was isolated according to the method described by Ghazi et al. (2019) [83]

For miRNA expression, cDNA was synthesized using the miScript II RT Kit (Qiagen, Hilden, Germany, 218161), as per the manufacturer’s instructions. The expression of miR-30c was analyzed using the miScript SYBR Green PCR Kit (Qiagen, Hilden, Germany, 218073) and the miR-30c primer assay (Qiagen, Hilden, Germany, MS00009366), as per the manufacturer’s instructions. Samples were amplified using the CFX96 Touch^TM^ Real-Time PCR Detection System (Bio-Rad, Hercules, CA, USA) with the following cycling conditions: initial denaturation (95 °C, 15 min), followed by 40 cycles of denaturation (94 °C, 15 s), annealing (55 °C, 30 s), and extension (70 °C, 30 s).

For mRNA expression, cDNA was prepared using the Maxima H Minus First Strand cDNA Synthesis Kit (Thermo-Fisher Scientific, Waltham, MA, USA, K1652), as per the manufacturer’s instructions. The expression of *KDM5B*, *PTEN*, *AKT*, and *CHK1* was determined using the Powerup SYBR Green Master Mix (Thermo-Fisher Scientific, Waltham, MA, USA, A25742), as per the manufacturer’s instructions. Samples were amplified using the CFX96 Touch^TM^ Real-Time PCR Detection System (Bio-Rad, Hercules, CA, USA) with the following cycling conditions: initial denaturation (95 °C, 8 min), followed by 40 cycles of denaturation (95 °C, 15 s), annealing (Temperatures: Table 1, 15 s), and extension (72 °C, 30 s).

Relative gene expression was determined using the method described by Livak and Schmittgen [84]. 2^−ΔΔCt^ represents the fold change relative to the untreated control. miRNA and mRNA of interest were normalized against the house-keeping genes, *RNU6* (Qiagen, Hilden, Germany, Ms000033740) and *GAPDH*, respectively.

### 5.5. Chromatin Immunoprecipitation Assay

H3K4me3 at the *PTEN* promoter region was determined using the chromatin immunoprecipitation (ChIP) assay. Histones were crosslinked to DNA by incubating (37 °C, 10 min) the cells in 37% formaldehyde. Cells were washed in cold 0.1 M PBS (containing protease inhibitors), mechanically lysed and centrifuged (2000 rpm, 4 °C, 4 min). The DNA pellet was re-suspended in sodium dodecyl sulphate (SDS)–lysis buffer (200 µL; 1% SDS, 10 mM Ethylenediaminetetraacetic Acid (EDTA), and 50 mM Tris; pH 8.1) and sheared by homogenization. Samples were centrifuged (13,000 rpm, 4 °C, 10 min) and supernatants were diluted with ChIP dilution buffer (0.01% SDS, 1.1% Tritonx-100, 1.2 mM EDTA, 16.7 mM Tris-HCl (pH 8.1), and 167 mM NaCl). The diluted supernatants were split into equal fractions. Anti-H3K4me3 (Abcam, Cambridge, UK, ab12209) was added to one fraction, while no antibody was added to its counterpart. Both fractions were incubated overnight at 4 °C. A 50% slurry of Protein A agarose and salmon sperm DNA (Merck, Kenilworth, NJ, USA, 16-157) was added to all samples and incubated (4 °C, 1 h) with gentle rotation. Thereafter, samples were centrifuged (1000 rpm, 4 °C, 1 min), and pellets were washed once with the following buffers: low salt immune complex wash buffer (0.1% SDS, 1% Tritonx-100, 2 mM EDTA, 20 mM Tris-HCl (pH 8.1), and 150 Mm NaCl), high salt immune complex wash buffer (0.1% SDS, 1% Tritonx-100, 2 mM EDTA, 20 mM Tris-HCl (pH 8.1), and 500 mM NaCl), Lithium chloride immune complex wash buffer (0.25 M LiCl, 1% IGEPAL, 1% deoxycholic acid, 1 mM EDTA, and 10 mM Tris; pH 8.1), and twice with TE buffer (10 mM Tris-HCl, 1 mM EDTA; pH 8.0). DNA was eluted using elution buffer (1% SDS, 0.1 M NaCHO_3_) for 15 min (gentle rotation, RT). Samples were centrifuged (1000 rpm, 4 °C, 1 min) and elution was repeated on the protein A agarose/ssDNA pellet. Eluates were combined and incubated in 5 M NaCl (65 °C, 4 h) to reverse crosslinks. DNA was further purified using a DNA Clean & Concentrator-5 kit, as per the manufacturer’s instructions (Zymo research, Irvine, CA, USA, D4003).

H3K4me3 immunoprecipitated chromatin was used in a RT-qPCR reaction (described in 2.3.) to determine H3K4me3 at the *PTEN* promoter (Sense: 5′- CGC CCA GCT CCT TTT CCC-3′; Anti-sense: 5′- CTG CCG CCG ATT CTT AC-3′). The fold enrichment method was used to normalize data obtained from the ChIP-qPCR.

### 5.6. Protein Isolation and Western Blotting

Protein was isolated using Cytobuster reagent (Merck, Kenilworth, NJ, USA, 71009-3) supplemented with protease and phosphatase inhibitors (Roche, Basel, Switzerland, 05892791001 and 04906837001, respectively). Cells were mechanically lysed, and centrifuged (13,000 rpm, 4 °C, 10 min). Supernatants were used to quantify protein concentration via the bicinchoninic acid assay (BCA). Proteins were standardized to 1 mg/mL. The expression of KDM5B (Abcam, Cambridge, UK, ab19884), H3K4me3 (Abcam, Cambridge, UK, ab12209), PTEN (Cell Signalling Technologies, Danvers, MA, USA, 9552S), p-ser473-AKT (Cell Signaling Technologies, Danvers, MA, USA, 9271S), AKT (Cell Signaling Technologies, Danvers, MA, USA 9272S), PI3K (Cell Signaling Technologies, Danvers, MA, USA, 4249S), p-ser280-CHK1 (Cell Signaling Technologies, Danvers, MA, USA, 23475), and CHK1 (Cell Signaling Technologies, Danvers, MA, USA, 2360S) were determined using Western blotting as previously described [43]. The Image Lab Software version 5.0 (Bio-Rad, Hercules, CA, USA) was used to measure band densities of expressed proteins. Protein expression is represented as relative band density and calculated by normalizing the protein of interest against the housekeeping protein, β-actin.

### 5.7. Statistical Analysis

All statistical analysis was performed using GraphPad Prism version 5.0 (GraphPad Software Inc., San Diego, CA, USA). The unpaired *t* test was used for all assays. One-way ANOVA with Dunnet’s post-test was used to evaluate the significant effect of FB_1_ in all Supplementary Figures. All results are presented as the mean ± standard deviation, unless otherwise stated. A value of *p* < 0.05 was considered to be statistically significant.

### 5.8. Ethics Approval 

Approval was received from the University of Kwa-Zulu Natal’s Biomedical Research Ethics Committee. Ethics number: BE322/19.

## Figures and Tables

**Table 1:** The annealing temperatures (°C) and primer sequences for the genes of interest.

Gene	Annealing Temperature (°C)	Primer	Sequence
*KDM5B*	55	Sense	5′-CGA CAA AGC CAA GAG TCT CC-3′
		Anti-sense	5′-CTG CCG TAG CAA GGC TATTC-3
*PTEN*	56.6	Sense	5′-TTT GAA GAC CAT AAC CCA CCA C-3′
		Anti-sense	5′-ATT ACA CCA GTT CGT CCC TTT C-3′
*AKT1*	55	Sense	5′-GCC TGG GTC AAA GAA GTC AA-3′
		Anti-sense	5′-CAT CCC TCC AAG CTA TCG TC-3′
*CHK1*	59.1	Sense	5′-CCA GAT GCT CAG AGA TTC TTC CA-3′
		Anti-sense	5′-TGT TCAACA AAC GCT CAC GAT TA-3′
*GAPDH*	Same as gene of interest	Sense	5′-TCCACCACCCTGTTGCTGTA-3′
		Anti-sense	5′-ACCACAGTCCATGCCATCAC-3′

## Supplementary Materials

The following are available online at https://www.mdpi.com/2072-6651/12/10/625/s1, Figure S1: The cytotoxic effects of FB_1_ on HepG2 cells, Figure S2: FB_1_ induced 8-OHdG levels in HepG2 cells, Figure S3: FB_1_ altered miR-30c expression in HepG2 cells, Figure S4: The effect of FB_1_ on KDM5B and H3K4me3 expression in HepG2 cells, Figure S5: FB_1_ induced KDM5B and miR-30c modulates PTEN expression, Figure S6: The effect of FB_1_ on the PI3K/AKT signalling cascade, Figure S7: The influence of FB_1_ on CHK1 expression in HepG2 cells. The additional supplementary material file includes results for cell viability and data generated using 5 and 100 µM.
