# Supplementary material for: Fumonisin B1 Epigenetically Regulates PTEN Expression and Modulates DNA Damage Checkpoint Regulation in HepG2 Liver Cells
Source: Toxins (Basel). 2020 Sep 30;12(10):625. doi: 10.3390/toxins12100625 (PMC7601513; doi:10.3390/toxins12100625)
Supplement: Supplementary file 1 [file toxins-12-00625-s001.pdf]

Supplementary Materials: Fumonisin B1 Epigenetically Regulates PTEN Expression and Modulates DNA Damage Checkpoint Regulation in HepG2 Liver Cells

Thilona Arumugam, Terisha Ghazi and Anil Chuturgoon

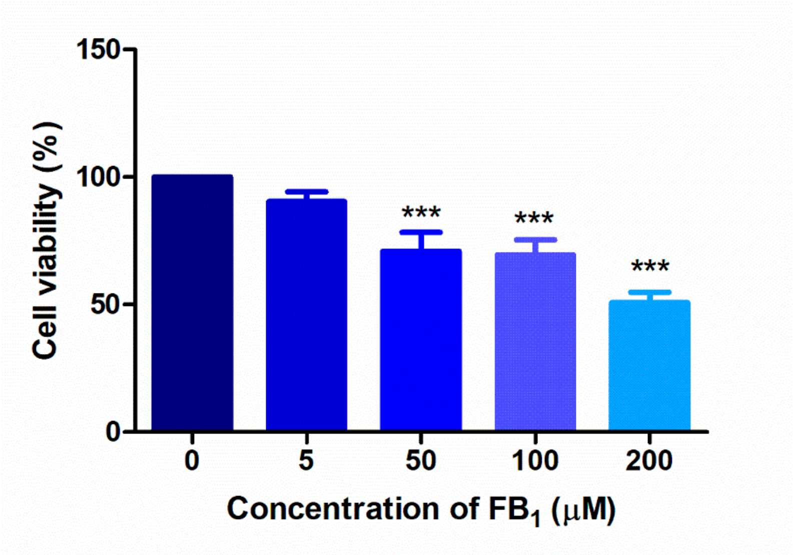

Figure S1. The cytotoxic effects of FB_1 on HepG2 cells. HepG2 cells were treated with 0, 5, 50, 100 and 200 μM FB_1 for 24h. Cell viability was determined using the crystal violet assay and expressed as a percentage of the untreated control. Control viability was taken as 100%. FB_1 significantly altered the cell viability of HepG2 cells. Data is represented as mean percentage cell viability ± SD (n = 3) (*** $p \leq 0.001$; one-way ANOVA with the Dunnet: compare all columns to control post-test).

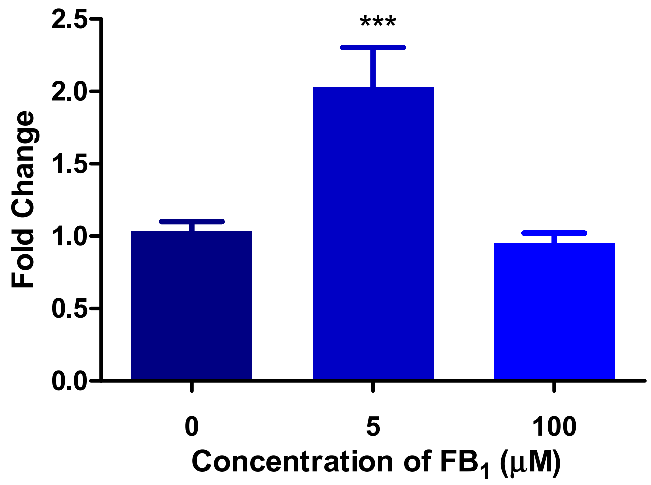

Figure S2. FB_1 induced 8-OHdG levels in HepG2 cells. 8-OHdG levels were measured as a marker of oxidative DNA damage. FB_1 significantly altered 8-OHdG levels in HepG2 cells (***$p = 0.0007$). Data is represented as mean fold change ± SD (n = 3) (*** $p \leq 0.001$; one-way ANOVA with the Dunnet: compare all columns to control post-test).

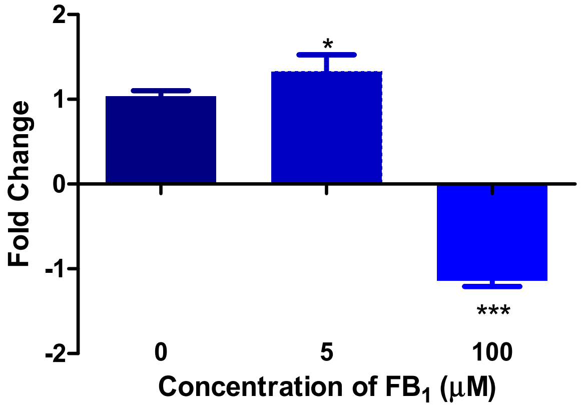

Figure S3. FB1 altered miR-30c expression in HepG2 cells. qPCR analysis of miR-30c showed that FB1 significantly altered miR-30c expression (***p < 0.0001). Results are represented as mean fold-change ± SD (n = 3) (*p < 0.05, ***p < 0.0001; one-way ANOVA with the Dunnet: compare all columns to control post-test).

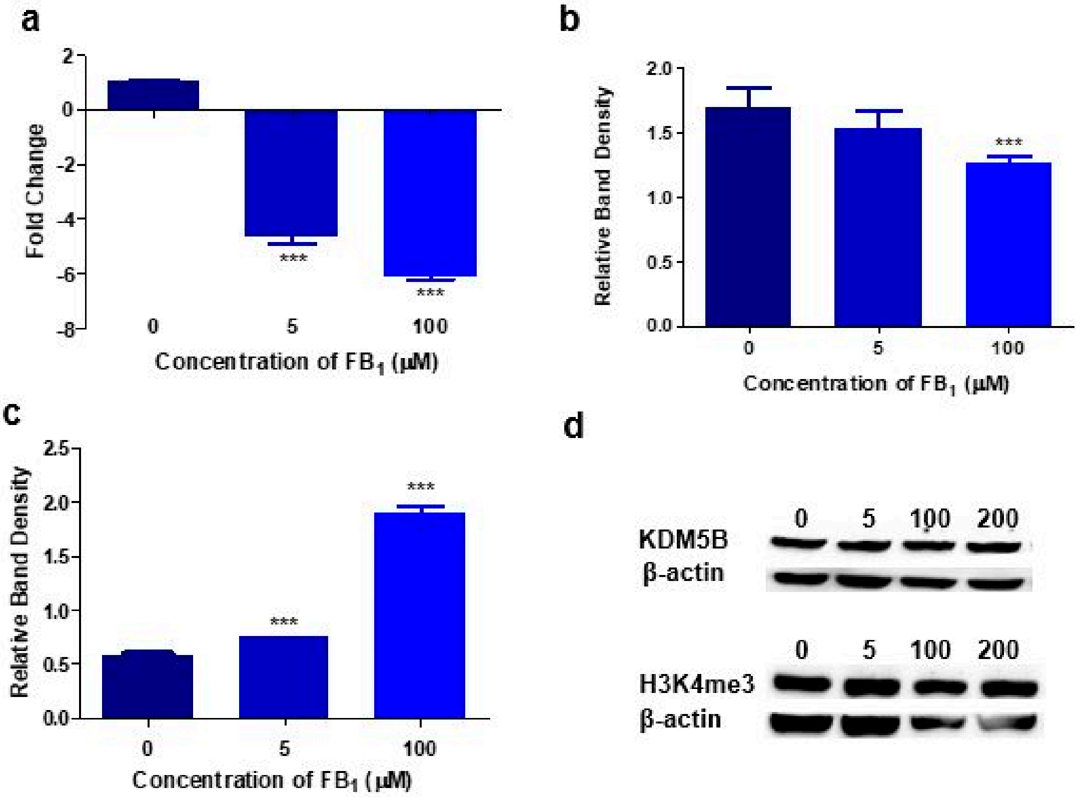

Figure S4. The effect of FB1 on KDM5B and H3K4me3 expression in HepG2 cells. FB1 reduced both the transcript (**a**; ***p < 0.0001) and protein (**b**; *p = 0.0106) expression of KDM5B. There was a dose-dependent increase in total H3K4me3 (**c**; *** p < 0.0001). Western blot images of KDM5B and H3K4me3 (**d**). KDM5B and H3K4me3 expression was normalized against β-actin. Results are represented as mean fold-change ± SD (n = 3) for gene expression and mean relative band density ± SD (n = 3) for protein expression (***p < 0.0001; one-way ANOVA with the Dunnet: compare all columns to control post-test).

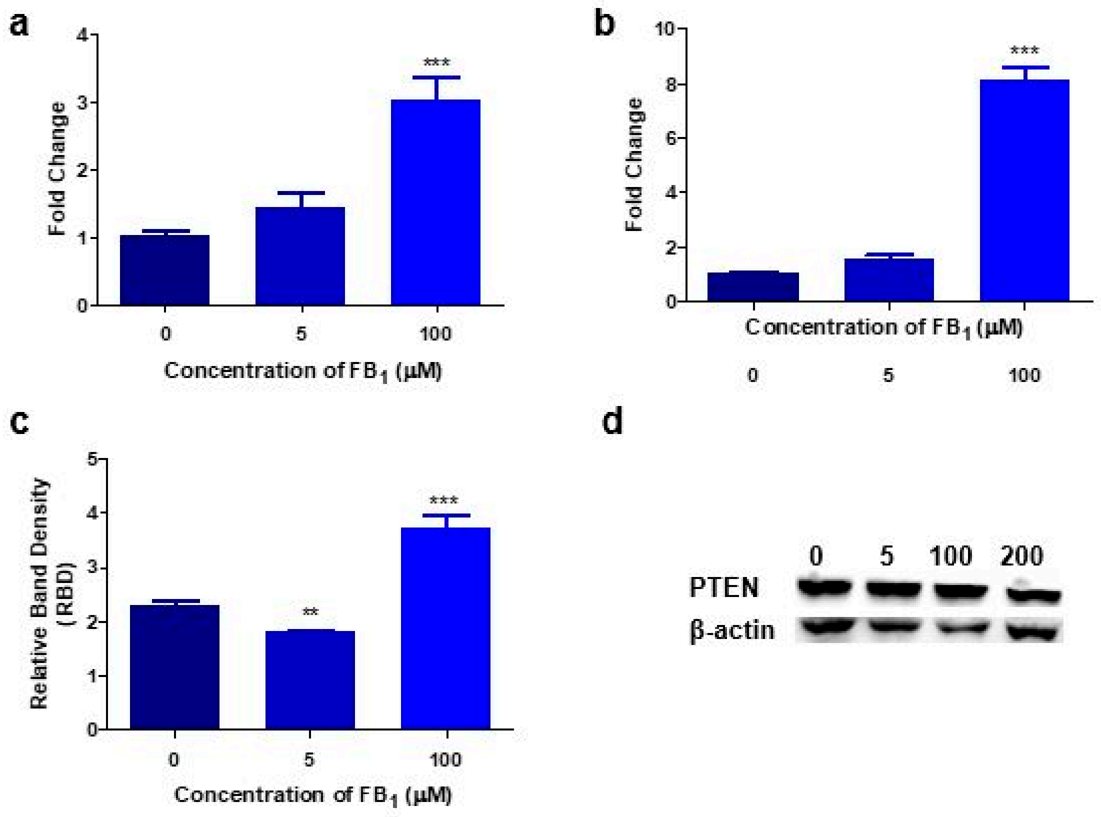

Figure S5. FB_1 induced KDM5B and miR-30c modulates PTEN expression. PTEN expression is under the influence of both KDM5B and miR-30c. **(a)** Low levels of KDM5B allowed for the increased H3K4me3 at *PTEN* promoter regions (*** $p < 0.0001$). **(b)** This resulted in significantly higher levels of *PTEN* transcripts (*** $p < 0.0001$). **(c)** However, miR-30c inhibited PTEN translation/protein expression at 5 µM FB_1 but increased PTEN translation at 100 µM FB_1 (*** $p < 0.0001$). **(d)** Western blot images of PTEN. PTEN expression was normalized against β-actin. Results are represented as mean fold-change ± SD (n = 3) for gene expression and mean relative band density ± SD (n = 3) for protein expression (*$p < 0.05$, ***$p < 0.0001$; one-way ANOVA with the Dunnet: compare all columns to control post-test).

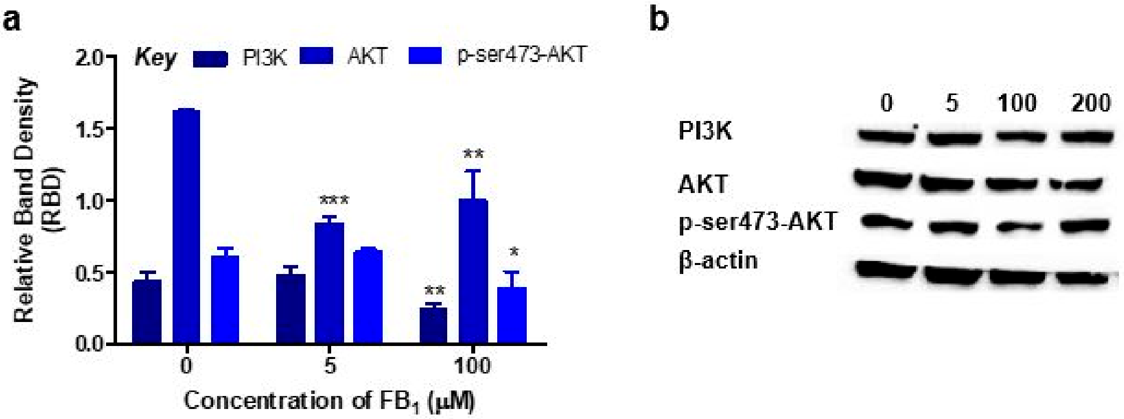

Figure S6. The effect of FB_1 on the PI3K/AKT signalling cascade. **(a)** Western blotting was used to determine the effect of FB_1 on the PTEN/PI3K/AKT signalling network. FB_1 significantly altered PI3K (*** $p < 0.0001$), AKT (*** $p = 0.0004$) and p-ser473-AKT (*$p < 0.0174$) protein expression. **(b)** Western blot images of PI3K, AKT and pAKT. p-ser473-AKT expression was normalized against AKT and

PI3K and AKT expression was normalized against β-actin. Data is represented as mean RBD ± SD (n = 3), (* $p \leq 0.05$, **$p \leq 0.01$ and ***$p \leq 0.001$; one-way ANOVA with the Dunnet: compare all columns to control post-test).

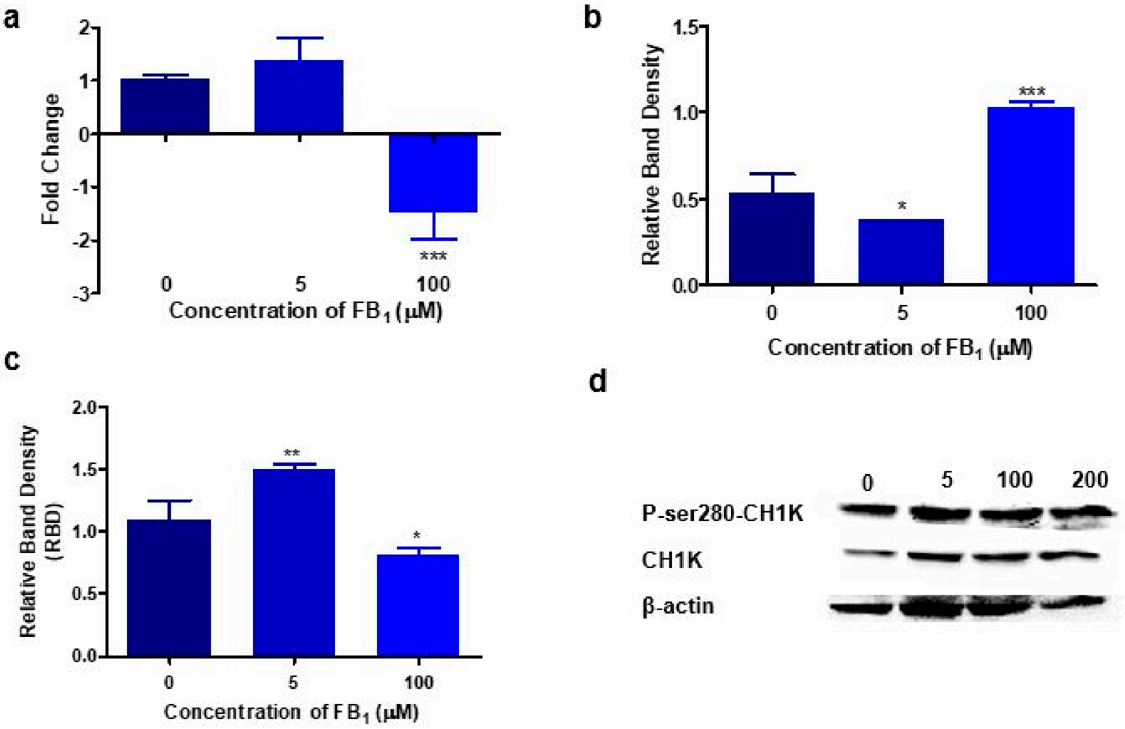

Figure S7. The influence of FB_1 on CHK1 expression in HepG2 cells. FB_1 significantly altered *CHK1* gene expression (**a**; ***$p = 0.0001$), CHK1 protein expression (**b**; *** $p < 0.0001$) and p-ser280-CHK1 (**c**; ***$p = 0.0006$). **(d)** Western blot images of CHK1 and p-ser280-CHK1. CHK1 expression was normalized against β-actin and p-ser280-CHK1 was normalized against CHK1. Gene expression is represented as fold changes ± SD relative to the control and protein expression is represented as mean RBD ± SD (*$p \leq 0.05$, **$p \leq 0.01$ and ***$p \leq 0.001$; one-way ANOVA with the Dunnet: compare all columns to control post-test).
